# Clemastine Promotes Differentiation of Oligodendrocyte Progenitor Cells Through the Activation of ERK1/2 *via* Muscarinic Receptors After Spinal Cord Injury

**DOI:** 10.3389/fphar.2022.914153

**Published:** 2022-07-05

**Authors:** Lu-Yao Tong, Yong-Bing Deng, Wei-Hong Du, Wen-Zhu Zhou, Xin-Yu Liao, Xue Jiang

**Affiliations:** ^1^ Department of Biochemistry and Molecular Biology, Molecular Medicine and Cancer Research Center, College of Basic Medicine, Chongqing Medical University, Chongqing, China; ^2^ Department of Chongqing Emergency Medical Center, Chongqing University Center Hospital, School of Medicine, Chongqing University, Chongqing, China

**Keywords:** clemastine, oligodendrocyte progenitor cells, ERK1/2, muscarinic receptor, myelin regulatory factor, oligodendrocyte transcription factor 2

## Abstract

The recovery of spinal cord injury (SCI) is closely associated with the obstruction of oligodendrocyte progenitor cell (OPC) differentiation, which ultimately induces the inability to generate newly formed myelin. To address the concern, drug-based methods may be the most practical and feasible way, possibly applying to clinical therapies for patients with SCI. In our previous study, we found that clemastine treatment preserves myelin integrity, decreases the loss of axons, and improves functional recovery in the SCI model. Clemastine acts as an antagonist of the muscarinic acetylcholine receptor (muscarinic receptor, MR) identified from a string of anti-muscarinic drugs that can enhance oligodendrocyte differentiation and myelin wrapping. However, the effects of clemastine on OPC differentiation through MRs in SCI and the underlying mechanism remain unclear. To explore the possibility, a rat model of SCI was established. To investigate if clemastine could promote the differentiation of OPCs in SCI *via* MR, the expressions of OPC and mature OL were detected at 7 days post injury (dpi) or at 14 dpi. The significant effect of clemastine on encouraging OPC differentiation was revealed at 14 dpi rather than 7 dpi. Under pre-treatment with the MR agonist cevimeline, the positive role of clemastine on OPC differentiation was partially disrupted. Further studies indicated that clemastine increased the phosphorylation level of extracellular signal–regulated kinase 1/2 (p-ERK1/2) and the expressions of transcription factors, Myrf and Olig2. To determine the relationship among clemastine, ERK1/2 signaling, specified transcription factors, and OPC differentiation, the ERK1/2 signaling was disturbed by U0126. The inhibition of ERK1/2 in SCI rats treated with clemastine decreased the expressions of p-ERK 1/2, Myrf, Olig2, and mature OLs, suggesting that ERK1/2 is required for clemastine on promoting OPC differentiation and that specified transcription factors may be affected by the activity of ERK1/2. Moreover, the impact of clemastine on modulating the level of p-ERK 1/2 was restricted following cevimeline pre-injecting, which provides further evidence that the role of clemastine was mediated by MRs. Altogether, our data demonstrated that clemastine, mediated by MRs, promotes OPC differentiation under the enhancement of Myrf and Olig2 by activating ERK1/2 signaling and suggests a novel therapeutic prospect for SCI recovery.

## Introduction

Spinal cord injury (SCI) impairs the functions of the spinal cord temporarily or permanently (Ahuja et al., 2017b; Courtine and Sofroniew, 2019; Liau et al., 2020). Its pathophysiology comprises initial mechanical trauma, and secondary injury cascades progressively permeabilized glia cells and neurons, which potently inhibit myelin regeneration (Gaudet and Fonken, 2018; Anjum et al., 2020). Accumulating studies have indicated that oligodendrocyte progenitor cells (OPCs) react rapidly to SCI in chronic demyelinating lesions, possessing capacities to compensate for oligodendrocyte (OL) loss and demyelination caused by SCI (Barnabé-Heider et al., 2010; Alizadeh and Karimi-Abdolrezaee, 2016). Furthermore, due to the limited self-repair ability of the OPCs, stimulating OPC differentiation to replace damaged oligodendrocytes is a promising approach for the recovery of SCI (Almad et al., 2011; Duncan et al., 2020). Based on this knowledge, it is indispensable for conducting drug-based methods to modulate the differentiation of OPCs for the therapy of SCI.

It is previously reported that a cluster of US Food and Drug Administration (FDA)–approved anti-muscarinic compounds have been screened, which effectively enhanced oligodendrocyte differentiation and remyelination, including the most effective compound, clemastine (Green et al., 2017). Clemastine is a muscarinic receptor (MR, muscarinic acetylcholine receptor) antagonist exhibiting anti-muscarinic properties and is also a typical, widely available antihistamine. To date, clemastine has shown its therapeutic potential in demyelinating injuries, such as multiple sclerosis (Green et al., 2017; Wooliscroft et al., 2019) and white matter injury (Wang et al., 2018). Similarly, our group has carried out research on the efficacy of clemastine on SCI in recent years. In our latest findings, we demonstrated that clemastine treatment on SCI enhances myelination and neurobehavioral recovery, also including the significant delay of axonal degeneration (Du et al., 2022). As for muscarinic receptors (MRs), they are G protein–coupled receptors (GPCRs) broadly distributed in the central nervous system (CNS). Not only do MRs mediate the metabotropic actions of acetylcholine (ACh) but also act as important drug targets for a series of CNS diseases including Alzheimer’s disease (AD), Parkinson’s disease, and schizophrenia (Bock et al., 2018). However, in SCI, it remains largely unknown whether clemastine promotes OPC differentiation *via* muscarinic receptors. Thus, a potent direct-acting muscarinic receptor agonist cevimeline, same as clemastine, which could cross the blood–brain barrier (Oleksak et al., 2021), was chosen in this study to disturb the effect of clemastine *via* MRs for exploring the underlying mechanism of clemastine in SCI.

Emerging evidences have shown an important role of the ERK/MAPK pathway in the regulation of OPC differentiation (Guardiola-Diaz et al., 2012; Dai et al., 2014; Gaesser and Fyffe-Maricich, 2016). In these studies, the number of differentiating OPCs declined after the pharmacologic inhibition of the ERK/MAPK signaling pathway in the primary OPC culture. As far as ERK1 and ERK2 are concerned, they are representative extracellular signal–regulated kinases in the members of the MAPK family, whose expression intuitively predicts the change of the ERK/MAPK pathway level. Therefore, based on the aforementioned conjecture that clemastine mediated by MR promotes OPC differentiation in SCI, we further hypothesized whether ERK1/2 signaling was involved in the course of OPC differentiation induced by clemastine and then observed if the differentiation of OPCs was affected under the condition of ERK1/2 inhibition. Accumulating evidence demonstrates that OPC differentiation and myelination have required intricate transcriptional regulation which features the dominant role of master transcriptional factors including Myrf and Olig2 (Emery et al., 2009; Mei et al., 2013; Li and Richardson, 2016; Duncan et al., 2017). Myrf can directly bind putative enhancer regions of myelin genes (for instance, *Mbp* and *Plp*) to induce their expression. In parallel, under the knockdown of Myrf in oligodendrocytes by RNA interference, the expressions of most CNS myelin genes were inhibited (Duncan et al., 2017). With regard to Olig2, it participates in not only the oligodendroglial development but also the remyelination in CNS demyelinating disease models (Sun et al., 2013; Tan et al., 2014). However, the expressions of Myrf and Olig2 in SCI lesions are unknown. To figure out the expressions of Myrf and Olig2 during OPC differentiation, we detected their levels following SCI especially with clemastine administration. Furthermore, combined with this finding that the expressions of Myrf and Olig2 were downregulated in the Cnp-Cre; ERK1/2 dKO mice (Ishii et al., 2014), the levels of Myrf and Olig2 were tested with the limitation of ERK1/2 signaling in this study to explore if potential connections among ERK1/2, Myrf, and Olig2 exist. Therefore, in this study, we constructed the SCI model to mimic the local injury of the spinal cord, then investigated the role of clemastine in OPC differentiation *via* the muscarinic receptor in SCI, and explored the underlying mechanism that clemastine enhanced OPC differentiation by stimulating ERK1/2 signaling along with the enhancement of Myrf and Olig2. Our results should contribute to the framework for exploring and elucidating the mechanisms of clemastine for recovery following SCI.

## Materials and Methods

### Animals

Adult female Sprague–Dawley (SD) rats (6–8 weeks of age, weighting 220 ± 20 g) were purchased from the Experimental Animal Center of Chongqing Medical University. All the rats were kept in breeding facility with free water and chow, also under the standard conditions, with the environmental temperature maintained at 22 ± 2°C, the humidity preserved at 55 ± 5%, and the 12 h light–dark cycle. All animal experiments and experimental procedures were carried out after the animals adapted to the rearing for 1 week in accordance with an approval from the Medical Research Ethics Committee of Chongqing Medical University (license no. SYXK (Su) 2018–0003). All efforts were made to minimize the number of animals used and their suffering.

### Spinal Cord Injury Model Induction

Spinal cord injury model induction was carried out as given below: 1) Each rat was deeply anesthetized by intraperitoneal injection of 1% pentobarbital (50 mg/kg, i. p.) after fasting for 12 h; 2) the rat was fixed on the operating table in a prone position and then the hair on their back was shaved. The T10 segment of the spinal cord was approached through the touch of the fingers referring to the principle of the most obvious T2 thoracic spinous process; 3) after disinfection with 75% alcohol on the back skin, an incision of about 4 cm, centered at the T10 segment, was made on the skin by the use of a scalpel (scalpel blade size at 23); 4) then, the muscles around the T10 position were cut with ophthalmic scissors to reveal the spinous process and spine; 5) the corresponding dorsal lamina was removed by use of micro tweezers and dead skin scissors, and the target spinal cord segment was exposed. 6) Next, the target segment was clamped with an arterial clip featured as 3.7 cm in total length to hold it for 30 s combining with the clamping thickness as 0.35 mm laterally. 7) Phenomena such as tail wagging and both hind limb twitching immediately appeared, which indicate that the construction of spinal cord injury model is successful. 8) The muscle and skin of post-trauma rats were sutured in turn, and then these animals were sent to the heating room to be looked after carefully until they were out of anesthesia. 9) About 3 hours after the injury, the animals were administered.

### Drug Delivery

According to the manufacturer’s protocol, clemastine (Cle, Cat:S1847, SelleckChem, Houston, TX) and U0126 (Cat:HY-12031, MCE, United States) were dissolved in dimethyl sulfoxide (DMSO) at 35 mg/ml and 50 mg/ml and then further diluted in a sterile saline solution. The final concentration of DMSO was controlled at 3% in the sterile saline solution. In addition, cevimeline (Cevi, Cat:S6432, SelleckChem, Houston, TX) was soluble to sterile saline at 25 mg/ml. The necessary dose of drugs could be administered in a total volume of 10 ml/kg by p. o. or i. p. injection (Diehl et al., 2001).

All rats were randomly assigned to the sham group; vehicle group; clemastine group (Cle); clemastine coupled with the cevimeline group (Cle + Cevi); cevimeline group (Cevi); and clemastine combined with the U0126 group (Cle + U1026). In the sham group, the spinal cord tissues of the rats were exactly intact, just only removing the corresponding lamina. The rats in the clemastine group were treated with clemastine daily at 10 mg/kg/day by gavage (Du et al., 2022) and in the vehicle group were treated with the equivalent volume of vehicle (3% DMSO in sterile saline solution), which differs from that the animals in the fourth group which were administered cevimeline (5 mg/kg/day) by i. p(Nakahara et al., 1988; Dawson et al., 1994) in advance at 30 min and then given clemastine (10 mg/kg/day) orally. Simultaneously, the rats in the cevimeline group were injected with cevimeline once daily at 5 mg/kg. As for the last group, the rats were pretreated with U0126 (30 mg/kg/day) (Ahnstedt et al., 2015) at 30 min *via* i. p. and then administered clemastine (10 mg/kg/day, p. o.). The dose of compounds mentioned above per day was determined by the body weight of these animals. The SCI models received these compound contents for 7 or 14 consecutive days.

### Tissue Processing

All rats were deeply anesthetized with 1% pentobarbital (50 mg/kg, i. p.) and then performed a blood flush transcardially with 0.01 mol/L phosphate-buffered saline (PBS; containing, in mmol/L, 8.3 Na_2_HPO_4_, 1.7 NaH_2_PO_4_, and 0.145 NaCl, pH 7.4) after the last drug infusion. The samples from half of the animals per group were quickly collected on ice and flash-frozen in dry ice before being stored at −80°C until processing to Western blotting and qRT-PCR detection. The remaining rats in each group were perfused with 4% paraformaldehyde (PFA) in 0.1 mol/L phosphate buffer (PB; containing, in mol/L, 0.083 Na_2_HPO_4_ and 0.017 NaH_2_PO_4_, pH 7.4) and then the spinal cords were collected and post-fixed with 4% PFA in 0.1 mol/L PB overnight. These tissues were dehydrated in 20%, 30% sucrose in 0.01 mol/L PBS in turn. Next, these spinal cords were embedded in an optimal cutting temperature compound (O.C.T. Compound, Cat:4583, SAKURA, United States) and then cut into serial frozen sections coronally (20 μm) on a cryostat microtome (MS 1850; Leica, Wetzlar, Germany) for immunofluorescence staining. Due to spatial distinction of the pathological progression of spinal cord injury, we focus on the spinal cord derived from 5 mm rostral to 5 mm caudal of the center of the injury site (Ek et al., 2010).

### Western Blot Analysis

Tissues were homogenized in ice-cold radioimmunoprecipitation assay (RIPA) lysis buffer (Cat:P0013B; Beyotime, China) including a protease inhibitor. To remove cellular debris, the extracts were centrifuged at 12,000 rpm for 18 min at 4°C, whose concentrations were measured by a BCA protein assay kit (Cat:P0010; Beyotime, China). After adding the SDS-PAGE sample loading buffer (Cat:P0015; Beyotime, China) to the protein supernatants and heating them at 99°C for 10 min, these samples were subjected to Western blotting. Equal amounts of protein (30 µg) were separated by sodium dodecyl sulfate-polyacrylamide gel electrophoresis in 6, 10, and 12% gels and transferred to polyvinylidene difluoride membranes (PVDF, Cat:ISEQ00010; Millipore, United States). After transferring, the membranes were blocked with 5% skim milk dissolved in TBST (0.1% Tween 20 in 0.01 mol/L TBS (Cat:G0001; Servicebio, China)) at room temperature for 2 h and then incubated with the primary antibodies according to the manufacturer’s protocol at 4°C overnight. The primary antibodies are shown as below: MBP (1:1000, Cat:83,683, Cell Signaling Technology, United States), NG2 (1:1000, Cat:MAB5384-I, Millpore, United States), P-ERK1/2 (1:1000, Cat:BS94044, Bioworld, China), T-ERK1/2 (1:1000, Cat:BS 1968, Bioworld, China), MYRF (1:1000, Cat:BS72874, Bioworld, China), OLIG2 (1:4000, Cat:13999-1-AP, Proteintech, China), and GAPDH (1:10,000, Cat:AF7021, Affinity, United States). After washing three times with TBST, the blots were incubated with the corresponding secondary antibodies for 1 h at 37°C. After rinsing in TBST, visualization was performed with an ECL Western blotting substrate (Cat:KF8005, Affinity, United States). The intensities of these protein brands were quantified by using ImageJ software (National Institutes of Health, United States).

### Immunofluorescence Staining

Immunofluorescence staining was performed as described previously (Wang et al., 2020). Floating tissue sections taken from the cryoprotective solution were washed three times with PBSTx (0.01 mol/L PBS containing 0.5% Triton-X 100), then blocked in 5% bovine serum albumin (BSA), and dissolved in PBSTx (0.5% Triton-X 100) for 1 h at 37°C. Next, these sections were incubated with the primary antibodies as MBP (1:500, Cat: MAB395, Millipore, United States) and NG2 (1:1000, Cat:MAB5320, Millipore, United States) at 4°C overnight. After washing in PBSTx (0.5% Triton-X 100), the sections were incubated for 2 h at room temperature with appropriate secondary antibodies. Secondary antibodies included the following: AlexaFluor-488- or AlexaFluor-568-conjugated secondary antibodies against rabbit, mouse, or rat (1:800; Invitrogen, United States). The nuclei were counterstained with 6-diamidino-2-phenylindole (DAPI) for 10 min. The antibodies described previously were prepared in 5% BSA in PBSTx. Fluorescence images were captured using a confocal laser-scanning microscope (Olympus, FV1000, Shinjuku, Tokyo) with excitation wavelengths appropriate for AlexaFluor-488 (488 nm), −596 (568 nm), or DAPI (380 nm). The quantitative method of fluorescence images was performed as previously described (Du et al., 2022). We obtained photos at ×400 magnification with a fluorescence microscope. Each rat had at least three slices, and four to five fields in each slice were selected. The number of NG2+ positive cells per unit area or the MBP density was calculated in each field of view. All quantifications were performed using Image-Pro Plus 5.0 (Media Cybernetics, Silver spring, MD, United States).

### Quantitative Real-Time PCR

Total RNA was isolated using the RNAeasy™ Animal RNA Isolation Kit (Cat:R0026, Beyotime, China) according to the manufacturer’s instructions, and then the concentration and purity of total RNA per sample were determined using an ultraviolet–visible light spectrophotometer (NanoDrop 2000; Thermo Scientific). Next, cDNA was synthesized using 500 ng of RNA, 2 μL 5x PrimeScipt RT Master Mix (Perfect Real Time) (Cat:RR360A, Takara, Japan), and RNase Free dH_2_O. Then, qPCR reactions were assembled using synthesized cDNA, TB Green Premix Ex TaqTM Ⅱ (Tli RNase plus) (2X) (Cat:RR820A, Takara, Japan), and 10 μm PCR primers diluted to 0.4 μm final concentration along with sterilized water through the Bio-Rad MJ Mini Option Real-Time PCR System. Glyceraldehyde phosphate dehydrogenase (GAPDH) was used as the reference gene for expression analysis of genes of interest, and the mRNA expression was assessed using the 2^−^^ΔΔCT^ method. Each reaction was set up in triplicate. All target gene primers used in this study are as follows: Myrf (sense: GAC​AGC​CTC​AAG​TCA​ACT​GGC​A, antisense: GAC​TGA​TGC​AGG​CCT​GAT​CTG​G); Olig2 (sense: AAG​CTC​TCC​AAG​ATC​GCC​AC, antisense: GGT​GAC​CCC​CGT​AAA​TCT​CG); GAPDH (sense: GAA​GGT​CGG​TGT​GAA​CGG​AT, antisense: CCC​ATT​TGA​TGT​TAG​CCG​GGA​T).

### Statistical Analysis

All experiments were repeated at least three times, and all data are presented as the mean ± SEM. The statistical significance of the differences between the groups was determined by using Graph Pad Prism 7 software (GraphPad Software, San Diego, CA, United States). Data were checked for normal distribution by using the Shapiro–Wilk normality test in advance, and then, the statistical difference between the groups was analyzed using Student’s unpaired, two-tailed *t-*test, or if the data were subject to homogeneity of variance, the one-way ANOVA method was performed followed by using Tukey’s post-hoc test. *p < 0.05, **p < 0.01, and ***p < 0.001 were considered to be statistically significant.

## Results

### Clemastine Promotes the Differentiation of OPCs *via* Muscarinic Receptors in the Spinal Cord Injury Model

**FIGURE 1:**
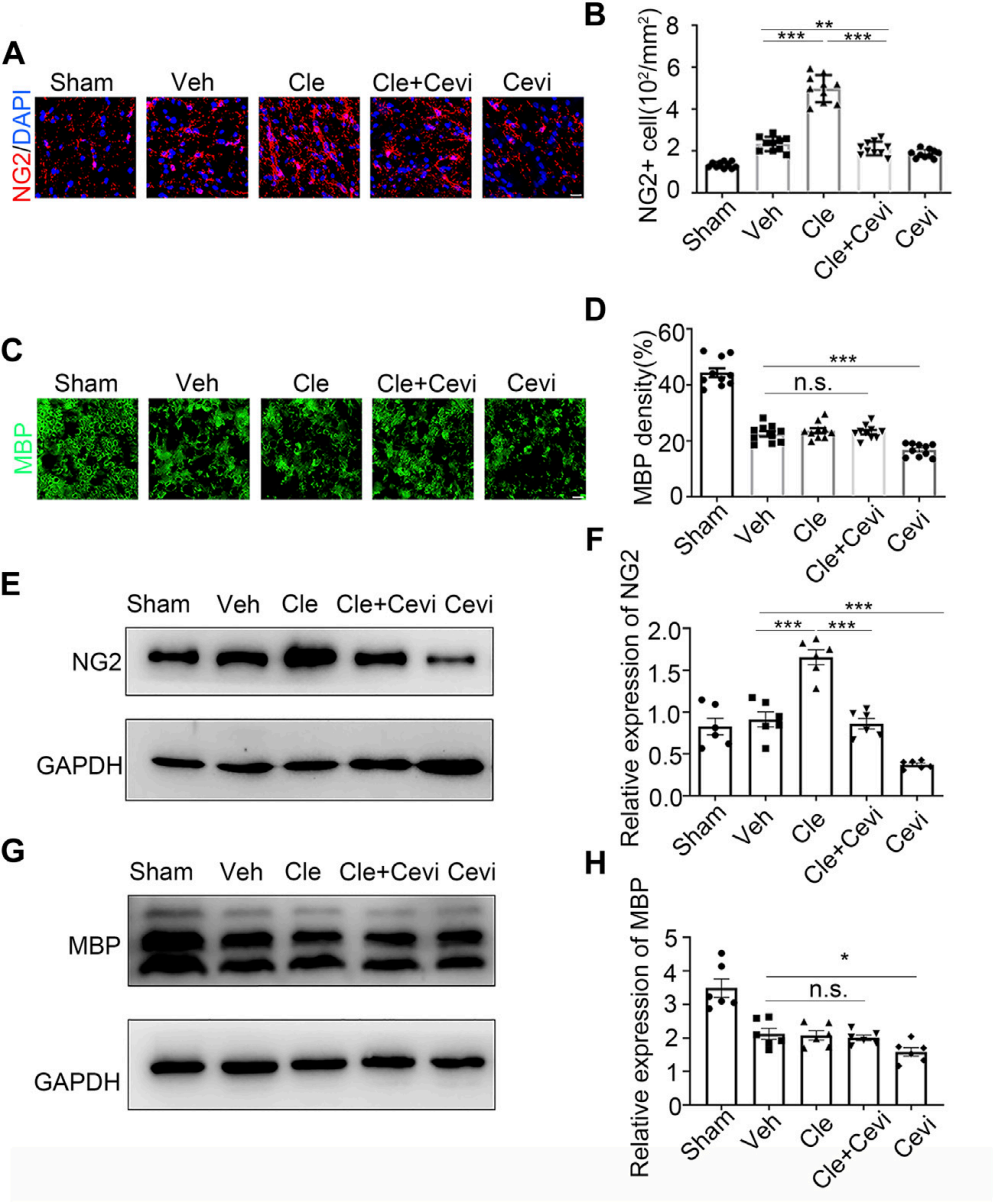
Effect of clemastine on oligodendrocyte progenitor cells at 7 dpi in rats. **(A)** Immunofluorescence staining for NG2 (red) displays oligodendrocyte precursor cells in the spinal cord white matter tracts in the Sham group, Veh group, Cle group, Cle + Cevi group, and Cevi group. DAPI (blue) was used as a nuclear counterstain. Scale bar, 50 µm **(B)** Number of NG2-positive cells in the spinal cord white matter tracts was quantified. **(C)** Representative images showing MBP (green) in the spinal cords from the Sham group, Veh group, Cle group, Cle + Cevi group, and Cevi group at 7 dpi. Scale bar, 50 µm **(D)** Quantification of MBP density in the spinal cord white matter tracts. **(E)** Expression of NG2 at 7 dpi was evaluated by Western blotting. GAPDH expression was served as an internal control. **(F)** Quantitative analysis of NG2 protein levels. **(G)** MBP protein expression was assessed by Western blot analysis. **(H)** Quantitative analysis of MBP protein levels. The protein levels of MBP were normalized to GAPDH. Data are shown as the mean ± SEM. *p < 0.05, **p < 0.01, ***p < 0.001 (*n* = 6 for each group at 7 dpi) was identified by the Student unpaired *t*-test or one-way ANOVA followed by Tukey’s post-hoc test.

**FIGURE 2:**
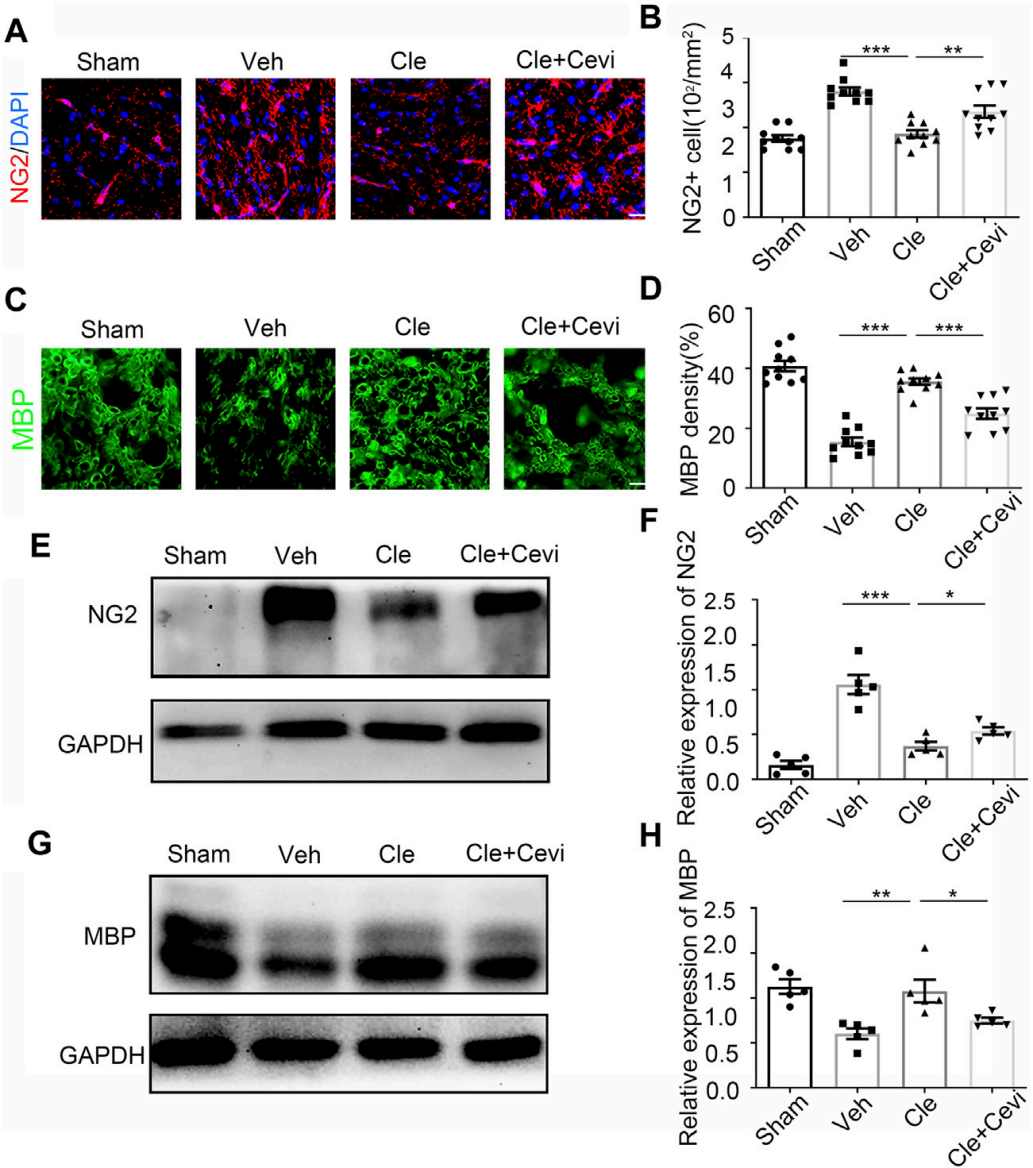
Clemastine promotes the differentiation of oligodendrocyte progenitor cells at 14 dpi in SCI. **(A,C)** Representative images showing NG2-positive OPCs **(A)** and area of MBP-positive mature OLs **(C)** in the spinal cords of Sham, Veh, Cle, and Cle + Cevi rats at 14 dpi. Scale bar, 50 µm. **(B,D)** Quantification of NG2+ cell numbers and MBP density from each group (*n* = 4). **(E,G)** Representative immunoblots of NG2 **(E)** and MBP **(G)** in the indicated situation. **(F,H)** Quantitative analysis of the expression of NG2 **(F)** and MBP **(H)** in the indicated situation. GAPDH expression from the same sample acts as an internal control. Error bars represent mean ± SEM, significance based on Student’s t-test with the respective controls (*p < 0.05, **p < 0.01, and ***p < 0.001, *n* = 5 for each group at 14 dpi).

To determine if the differentiation of OPCs is significantly promoted by clemastine in the SCI model, we took advantage of the spinal cord samples collected from these rats at 7 dpi. We identified oligodendrocyte progenitor cell maker as neural/glial antigen 2 (NG2) and mature oligodendrocyte marker as myelin basic protein (MBP) to detect the number of OPCs and the ratio of OLs in the spinal cord. The number of NG2-positive OPCs increased remarkably 7 days after treatment with clemastine at 10 mg/kg per day as compared to the vehicle group [Figures 1A,B, t (18) = 11.41, *p* < 0.0001], whereas clemastine had no significant effect on MBP density [Figures 1C,D, vehicle group = 22.53 ± 0.9721; Cle group = 23.65 ± 0.9288; Cle + Cevi group = 23.15 ± 0.7731; F (2,27) = 0.3943, *p* = 0.6780] yet. Meanwhile, the expression of NG2 protein was significantly enhanced in the spinal cord treated with clemastine when compared to the vehicle group [Figures 1E,F, t (10) = 5.858, *p* = 0.0002]. In addition, the level of MBP protein was similar to that of the vehicle controls [Figures 1G,H, vehicle group = 2.125 ± 0.1639; Cle group = 2.077 ± 0.1398; Cle + Cevi group = 2.099 ± 0.08182, F (2,15) = 0.1940, *p* = 0.8257]. The aforementioned phenomena may be reasonably explained, considering that most “new-born” OPCs at 1 week after SCI were undergoing the proliferative phase and did not yet differentiate into MBP-positive OLs (Hassannejad et al., 2019; Li et al., 2021). We extended the observation time properly and tried determining the effect of clemastine on OPC differentiation at 14 dpi. Compared to the vehicle group, the density of MBP + cells raised dramatically [Figures 2C,D, t (18) = 11.26, *p* < 0.0001] whose upward trend is entirely opposite to the tendency of the number of NG2+ cells [Figures 2A,B, t (18) = 7.924, *p* < 0.0001] in rats treated with clemastine. Consistent with these findings, the protein level of MBP was upregulated significantly [Figures 2G,H, t (8) = 3.424, *p* = 0.0090] in the clemastine group, while that of NG2 was reduced notably [Figures 2E,F, t (8) = 5.867, *p* = 0.0004]. These results suggest that clemastine has a positive influence on motivating OPC differentiation, possibly accelerating OPCs into the recruitment status in the early stages of injury (such as 7 dpi in this study).

To investigate whether the ability of clemastine in the SCI model to encourage OPC differentiation was mediated by MRs, cevimeline acts as a muscarinic receptor agonist to interfere with the impact of clemastine in SCI. Given that previous findings have identified that the MR acts as a negative regulator for oligodendroglial differentiation (Mei et al., 2016; Wang et al., 2018), the rats were solely administered with cevimeline (5 mg/kg, i. p.) for 7 consecutive days. The expressions on NG2 and MBP in animals treated with cevimeline were quantified to be decreased prominently as compared to the vehicle group [Figures 1E,F, t (10) = 5.890, *p* = 0.0002; Figures 1G,H, t (10) = 2.592, *p* = 0.0269]. In parallel, the expressions on mature OLs [Figures 1C,D, t (18) = 4.819, *p* = 0.0001] and OPCs [Figures 1A,B, t (18) = 3.764, *p* = 0.0014] in the Cevi group were also significantly lower than those in the vehicle group. Combined with the effect of cevimeline, the percentage of NG2+ cells [Figures 1A,B, t (18) = 12.40, *p* < 0.0001] and the NG2 protein levels [Figures 1A,B, t (10) = 7.286, *p* < 0.0001] in Cle + Cevi animals were significantly decreased compared to Cle animals at 7 dpi. Meanwhile at 14 dpi, compared to the Cle group, the density’s quantification of [Figures 2C,D, t (18) = 5.173, *p* < 0.0001] and the protein level [Figures 2G,H, t (8) = 2.543, *p* = 0.0345] of MBP emerged with a descending tendency; however, the expression of OPCs was higher in the Cle + Cevi group [Figures 2A,B, t (18) = 3.14, *p* = 0.0057; Figures 2E,F, t (8) = 2.669, *p* = 0.0284]. The aforementioned data demonstrated that clemastine works to enhance OPC differentiation through muscarinic receptors in the SCI model.

### Clemastine Might Enhance the Differentiation of Oligodendrocyte Progenitor Cells by Upregulating the Phosphorylation Level of ERK 1/2 *via* Muscarinic Receptors

**FIGURE 3:**
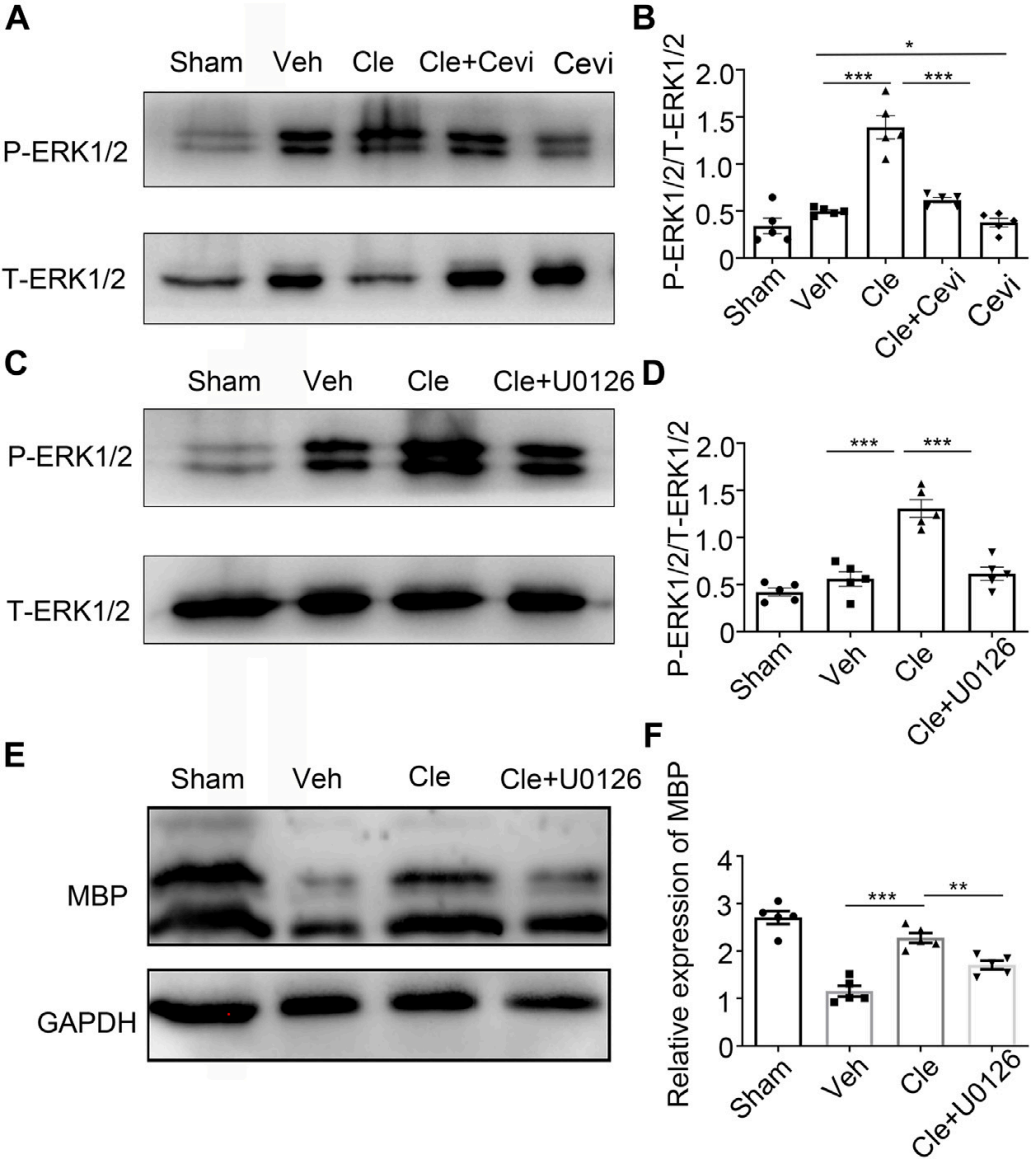
Clemastine promotes OPC differentiation through the upregulation of p-ERK1/2 *via* MR. **(A,C)** Protein levels of phosphorylated ERK1/2 and total ERK1/2 were accessed by Western blotting. **(B,D)** Intensities of p-ERK1/2 were quantified. Total ERK1/2 expression from the same sample acts as an internal control. **(E)** Representative Western blots of MBP were performed. **(F)** Quantitative analysis of protein levels of MBP. The MBP protein levels were normalized to GAPDH. Error bars represent the mean ± SEM (*n* = 5 for each group). *p < 0.05, **p < 0.01, and ***p < 0.001 by Student’s t-test with the respective controls.

Considering that ERK1/2, a crucial part for myelination, is essential for OPC demonstration to be OLs (Gonsalvez et al., 2016), we examined whether clemastine enhances the phosphorylation level of ERK 1/2 during the phase of OPC differentiation. The protein expression of p-ERK1/2 increased effectively (Figures 3A,B) in the clemastine (10 mg/kg) group, compared with the vehicle group [t (8) = 7.179, *p* < 0.0001]. To confirm whether cevimeline by itself blocked the level of p-ERK1/2, we found that the expression of P-ERK1/2 in the Cevi group was notably declined [Figures 3A,B, t (8) = 2.416, *p* = 0.0421] in comparison with the vehicle group. To measure whether these effects were associated with MR, spinal cords from the cevimeline (5 mg/kg)+clemastine rats were analyzed by immunoblotting the p-ERK1/2 protein level, and the p-ERK1/2 intensity (Figures 3A,B) was downregulated significantly as compared to the clemastine (10 mg/kg) group [t (8) = 6.119, *p* = 0.0003]. To assess whether there are changes in OPC differentiation under the disturbance of ERK1/2 activity through U0126, the levels of p-ERK1/2 and MBP {Figures 3C,D [t (8) = 5.952, *p* = 0.0003; t (8) = 4.157, *p* = 0.0032]} in the Cle + U0126 group, revealed by Western blotting, were significantly downregulated in comparison to the clemastine group. These results suggest that clemastine motivates the phosphorylation level of the ERK 1/2 *via* MR; in addition, OPC differentiation was affected under ERK1/2 inhibition.

### Expressions of Myrf and Olig2 Are Improved by Clemastine However Affected by ERK1/2 Activity *In Vivo*

**FIGURE 4:**
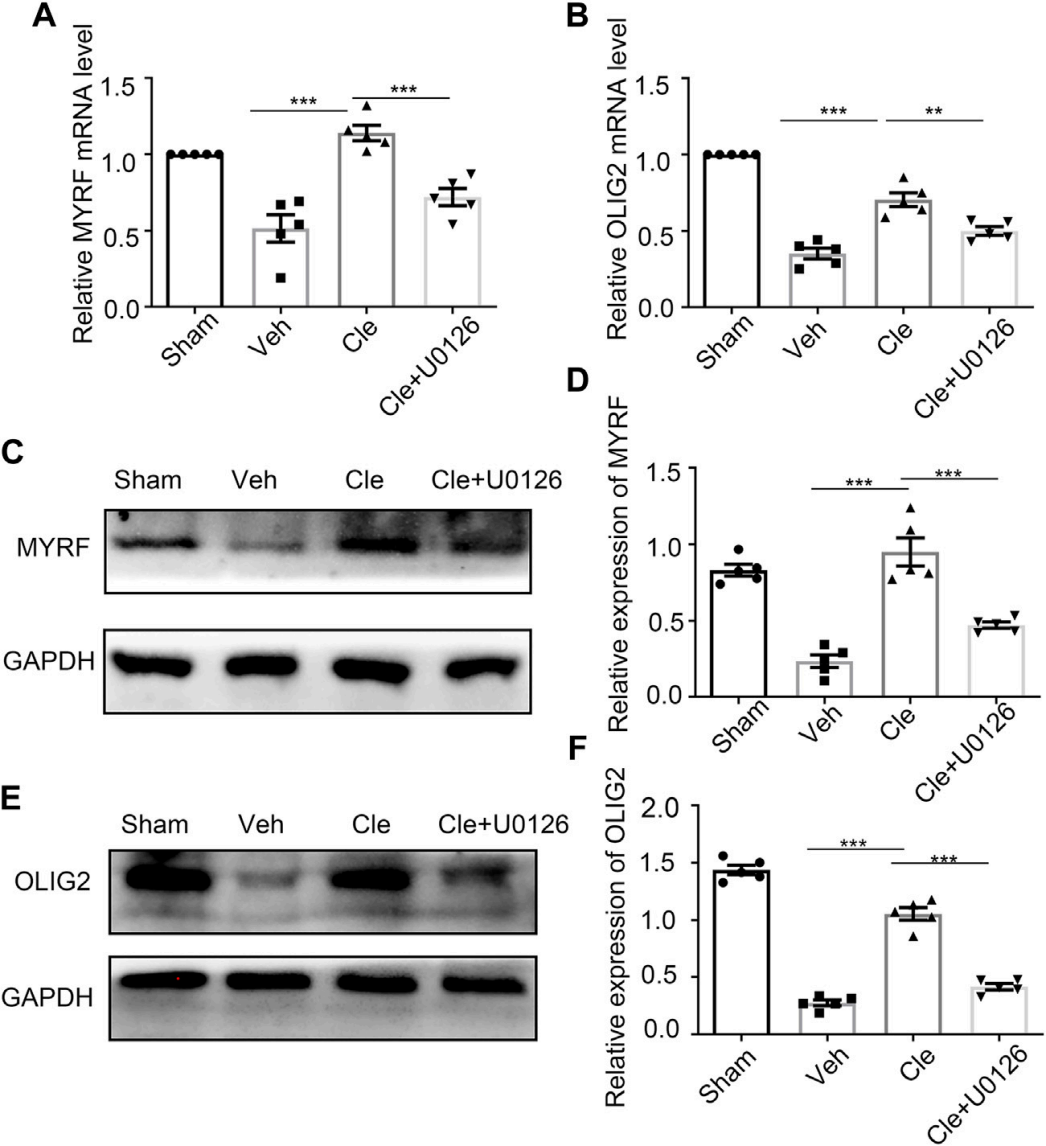
Influences of ERK1/2 activity on Myrf and Olig2 expression **(A,B)** qRT-PCR analysis of Myrf **(A)** and Olig2 **(B)** expression in the spinal cords from the Sham, Veh, Cle, and Cle + U1026 group. **(C,E)** Western blot analysis of Myrf **(C)** and Olig2 **(E)** was evaluated. The expression of Myrf and Olig2 is normalized to GAPDH expression. **(D,F)** Quantitative analysis of the protein levels of Myrf and Olig2. *p < 0.05, **p < 0.01, and ***p < 0.001 (*n* = 5 for each group).

It has been previously shown that Myrf and Olig2 are essential transcriptional factors that act as positive regulators in modulating the specification and differentiation of OLs (Yue et al., 2006; Mei et al., 2013; Küspert and Wegner, 2016). We tried to determine whether there were alterations of Myrf and Olig2 in these rats after SCI at 14 dpi. Spinal cords from the clemastine (10 mg/kg), sham, and vehicle groups were examined by qRT-PCR and Western blotting to identify the changes in Myrf and Olig2. As expected, clemastine can significantly boost Myrf and Olig2 mRNA levels [Figure 4A, t (8) = 6.061, *p* = 0.0003; Figure 4B, t (8) = 6.137, *p* = 0.0003] and their protein expressions [Figures 4C,D, t (8) = 7.098, *p* = 0.0001; Figures 4E,F, t (8) = 12.83, *p* < 0.0001] compared to the vehicle group. Given that clemastine can upregulate the phosphorylated ERK1/2 levels in the SCI model (present data), we investigated whether Myrf and Olig2, transcription factors known to regulate oligodendrocyte lineage cells, are possibly related to the downstream signaling of the ERK1/2 signaling cascade. Rats were pretreated with U0126 (30 mg/kg, i. p.) to inhibit the ERK1/2 signaling before clemastine administration, and then our hypothesis described previously was tested. The qRT-PCR results revealed that the mRNA levels of Myrf and Olig2 were significantly declined [Figure 4A, t (8) = 5.496, *p* = 0.0006; Figure 4B, t (8) = 3.851, *p* = 0.0049] as well as their protein signal intensities [Figures 4C,D, t (8) = 5.081, *p* = 0.0010; Figures 4E,F, t (8) = 10.53, *p* < 0.0001] in the Cle + U0126 rats compared with the clemastine group, which possibly means that these connections have existed between ERK1/2, Myrf, and Olig2 during the status of OPC differentiation. Combined with these findings, our results indicate that the expressions of Myrf and Olig2 could be stimulated by clemastine and simultaneously be obstructed with the interference of ERK1/2 signaling. Therefore, clemastine could boost the expressions of Myrf and Olig2 in SCI; moreover, these specified transcriptional factors (Myrf and Olig2) may be associated with the downstream signaling of the ERK1/2 signaling cascade.

## Discussion

Spinal cord injury (SCI) belongs to neurotraumatic events in the CNS under devastating conditions (Ahuja et al., 2017a; Ahuja et al., 2017b; Anjum et al., 2020). SCI-induced spinal cords are featured by a series of pathological processes, including oligodendrocyte loss and demyelination (Dimitrijevic et al., 2015; Yılmaz and Kaptanoglu, 2015). Myelin integrity, considered key to CNS physiology, is indispensable for its functional restoration after SCI (Papastefanaki and Matsas, 2015). Mature oligodendrocytes (OLs) are the myelinating cells in the CNS, which express myelin proteins such as MBP and are generated from a cohort of OPC developmental steps. Furthermore, the multilayered myelin sheaths were composed of mature OLs wrapping adjacent neuronal axons (Wittstatt et al., 2019). Therefore, promoting the differentiation from OPCs into mature OLs may contribute to remyelination (Patel and Klein, 2011). In this study, we focused on the strategies to promote OPC differentiation of SCI recovery.

### The Targeted Receptor Subtypes of Clemastine and Cevimeline

Accumulating evidence has shown that clemastine targets multiple muscarinic acetylcholine receptors (mAChR, muscarinic receptors) including M1R–M5R and histamine receptors. Despite the fact that antagonism of M1R can enhance myelination, it is not clear yet whether clemastine’s effect on SCI solely depends on M1R. mAChRs are involved in the metabotropic actions of acetylcholine (ACh) throughout the CNS, also critically situated as potential targets for treating multiple CNS disorders and influencing complex behaviors, such as cognition and motivation (Lebois et al., 2018). All mAChR subtypes possess at least one allosteric binding site that could be specifically targeted by chemical compounds (mostly small molecules). With the binding of these allosteric modulators, the affinity and/or efficacy of orthosteric (ACh) ligands were affected at the same time (Bock et al., 2018). In addition, the cholinergic system can be therapeutically affected by either direct-acting or indirect-acting cholinergic agents (Oleksak et al., 2021). However, generating ligands with a high degree of selectivity at each muscarinic receptor subtype has been exceptionally challenging (Thomsen et al., 2018). Recent efforts in high-throughput screening have identified several compounds capable of enhancing differentiation and myelination of oligodendrocytes. Included in this screening is clemastine; clemastine is a muscarinic receptor antagonist that has the most potential for enhancing oligodendrocyte differentiation and membrane wrapping (Green et al., 2017). Clemastine rescues myelination defects and promotes functional recovery following chronic hypoxic brain injury in mice (Cree et al., 2018). Moreover, in socially isolated mice, clemastine resulted in epigenetic changes in the oligodendrocytes but not in the neurons of the prefrontal cortex and enhanced OPC differentiation (Liu et al., 2016), additionally contributing to alleviation of a stress-related depressive-like phenotype (Su et al., 2018). Currently, clemastine exhibits high affinity to MRs through a receptor-binding assay of anti-muscarinic effects. It has been reported that clemastine is a MR antagonist, possibly possessing higher affinity toward the M1/M3 receptor than other receptor subtypes (Kubo et al., 1987); in further analyses, the target receptor(s) of clemastine was identified in the OPC culture from M1R–M5R knockout mice and then, the effects of the anti-muscarinic compounds were abolished in MR1-knockout mice, inducing similar numbers of OPCs and differentiated OLs compared to the vehicle-treated M1R-knockout mice, possibly suggesting that M1R could be the most potential mediator of the effects of clemastine on oligodendroglia (Mei et al., 2016), and then the role of clemastine in M1R KO rats with SCI was considered to be explored in our future studies. Cevimeline is a parasympathomimetic and direct-acting MR agonist, usually indicated for treating the symptoms of dry mouth in patients with Sjögren’s syndrome and the therapeutic remedy for treating AD (Oleksak et al., 2021). The toxicity of cevimeline in overdose has not been reported in the medical literature, whereas ingesting high doses of cevimeline (e.g., 30 mg/kg, p. o.) may still have side effects (parasympathomimetic effects), such as nausea, vomiting, and headache (Voskoboynik et al., 2011). Several studies have demonstrated that cevimeline is a direct-acting cholinergic agonist that inclines to bind and activate M1 and M3 subtypes of muscarinic receptors; moreover, the selectivity of cevimeline toward mAChR was evaluated by the EC_50_ values. This evaluation confirmed that the selectivity of cevimeline toward M3 was 2-fold lower, toward M5 was 3-fold lower, and toward the M4 receptor was 43-fold lower; in addition, its selectivity toward the M2 receptor was 46-fold lower than the selectivity for the M1 receptor, suggesting that cevimeline may be a potent M1R agonist (Heinrich et al., 2009). Combined with this knowledge about the type of potential binding receptor and the ability to cross the blood–brain barrier, both clemastine and cevimeline were considered a priority to inhibit the effect of the muscarinic receptor antagonist toward MRs. Here, the role of clemastine in SCI was investigated, and further cevimeline was chosen to verify if the effect of clemastine was mediated by muscarinic receptors in SCI. Our results indicate that OPC differentiation was enhanced with the treatment of clemastine, whereas in the case of pretreating cevimeline before administering clemastine in SCI rats, the course of OPCs to be differentiated OLs was partially blocked. Moreover, while these animals were injected by cevimeline only, these phenotypes about OPC development were obstructed, possibly suggesting that M1 agonism is unbeneficial for OPC differentiation following SCI.

Endogenous acetylcholine (ACh) acts as a vital neurotransmitter toward muscarinic receptors in the CNS, influencing learning and memory (Oleksak et al., 2021). Considering that the level of endogenous ACh is high enough to activate MRs, the effect of cevimeline in our study blocked on these phenotypes about OPC differentiation following SCI, indicating that endogenous Ach is not maximal possibly at 7 dpi in this study, in proof of the notion that the limitation of the effects of clemastine was induced by MR agonists to MRs. As previous studies have demonstrated, endogenous ACh showed a generalized decrease from 2.5 h until 4 days post-trauma in the center, periphery, and adjacent regions of the homolateral hemisphere in traumatic brain injury at an early period (such as 2.5 h, 1, and 4 days) (Scremin et al., 2006); meanwhile, intraperitoneal administration of cevimeline induces a significant decline of ACh content in the brain (Ogane et al., 1990). Thus, our attention mainly focuses on investigating if the role of clemastine was mediated by muscarinic receptors, and the purpose of interfering to the clemastine antagonistic effect on receptors has been achieved when pretreating cevimeline, so whether the binding type of clemastine toward muscarinic receptors is competitive or non-competitive, the interaction of cevimeline or clemastine to the receptors may not possibly be affected. Also at present, no scholars have researched on the binding type or site of clemastine toward muscarinic receptors. Given that cevimeline activates MR through intraperitoneal injection, we could not completely exclude the possibility that the effect of cevimeline may possess off-target effects or some other manner to contribute to the attenuation of OPC development in SCI. In addition, we are interested in exploring more information about endogenous Ach and the binding type of clemastine toward muscarinic receptors following SCI in further study.

### The Potential Mechanism of Clemastine in Promoting OPC Differentiation

Concerning the underlying mechanism on the effect of clemastine in promoting OPC differentiation, we illustrated that clemastine enhanced the phosphorylation level of ERK 1/2 partly through the muscarinic receptor and that the ERK 1/2 inhibitor U0126 blocked the p-ERK1/2 expression and the differentiation of OPCs. Associated with this, the prominent role of ERK1/2 in regulating OPC differentiation and myelin thickness expansion was revealed by analyzing the data from the deletion of Erk1/2 *in vitro or in vivo* (Fyffe-Maricich et al., 2011; Gaesser and Fyffe-Maricich, 2016). Erk1/2 dKO mice manifested apparent hypomyelination accompanied by decreases in mRNA and protein expressions of the myelin gene (Ishii et al., 2012). With sustaining activation of ERK1/2 to accelerate myelin repair after injury, myelin thickness was upregulated (Almad et al., 2011; Fyffe-Maricich et al., 2013). To assume that the enhancement of ERK1/2 signaling induced by clemastine mainly occurs in oligodendroglia, our result demonstrated that the expression of p-ERK1/2 in the Cle group was higher than that in the vehicle group (Figures 3A,B). Notably, the infiltration of T-cells and macrophages and the activation of microglia in demyelinated lesions in SCI or experimental autoimmune encephalomyelitis (EAE) were examined; however, there is no significant difference between the vehicle and clemastine groups, suggesting that the action of clemastine does not change the inflammatory response in the aspect of cellular infiltration (Mei et al., 2016; Du et al., 2022). As we all know, microglia and astrocytes are the major sources of CNS inflammatory response; therefore, we could assume that clemastine may not be involved in the adjustment of microglia or astrocytes. With that said, clemastine notably suppressed microglial activation and ameliorated astrocytic loss in a mouse model of depressive-like behavior (Su et al., 2018), indicating that there is no relationship between the mechanism of clemastine and the activation of microglia and astrocyte.

A cohort of master transcriptional factors, including Myrf and Olig2, regulates myelin generation in the CNS (Li and Richardson, 2016). The full length of the Myrf protein autoproteolytically cleavages into a membrane-bound C-terminal region and a nuclear-targeted N-terminal fragment. Using chromatin-immunoprecipitation-sequencing (ChIP-Seq), the finding demonstrated that the N-terminal cleavage product directly binds the regulatory regions of several myelin-associated genes, such as *Mbp*, *Plp*, and *Mag* (Bujalka et al., 2013). The deletion of Myrf from OPCs resulted in severely impaired remyelination in both the spinal cord and corpus callosum. Meanwhile, these OPCs, in response to demyelination, showed less ability to express myelin proteins (Duncan et al., 2017). Olig2, a member of the basic helix-loop-helix (b-HLH) family (Li and Richardson, 2016), regulates the formation and generation of oligodendrocyte lineage cells (Zhou and Anderson, 2002; Tan et al., 2017). Moreover, previous reports have shown that Olig2 participates in not only the OL development stages but also in myelin repair in MS models (Sun et al., 2013; Tan et al., 2014; Tomas-Roig et al., 2016), although the exact role of Olig2 in CNS demyelinating models remains controversial according to recent evidences (Buffo et al., 2005; Hu et al., 2012). Considering that Myrf plays a critical role in the transition of oligodendrocytes from premyelinating to be myelinating and was expressed in new oligodendrocytes in genetically fate-mapped OPCs following lysolecithin-induced demyelination of the corpus callosum in mice (Duncan et al., 2017), additionally, the ablation of Olig2 in OPCs significantly inhibits differentiation, resulting in hypomyelination (Mei et al., 2013); thus, our efforts focus on the expressions of Myrf and Olig2 at the phase of OPC differentiation. Based on this argument, in our study, the spinal cords from rats at 14 dpi were taken to detect the changes of Myrf and Olig2 during the recovery of SCI, and we found that both levels were clearly enhanced in Cle animals as compared to vehicle animals, indicating that the expressions of Myrf and Olig2 were reduced following SCI but reversed by clemastine. Similarly, in a preliminary test, there is no statistical difference in the expressions of Myrf and Olig2 at 7 dpi between vehicle and Cle animals.

In further research, the levels of both Myrf and Olig2 were significantly declined under the disruption of ERK1/2 activity, which implies there might exist potential relationships among ERK1/2, Myrf, and Olig2. Emerging evidences have shown that the results of the qRT–PCR analysis of Myrf and Olig2 declined from *Cnp-Cre;Erk1/2 dKO* mice at the postnatal age of 20 days (Ishii et al., 2014), especially Myrf expression, when compared to the controls. Similarly, the mRNA level of Myrf was restored to normal through the enhancement of ERK1/2 activity in *FGF-Receptor-type-2 (FGFR2)–*deficient mice, whereas myelin thickness was regulated by activation of ERK1/2-MAPK medicated by FGFR2 (Furusho et al., 2017).

## Conclusion

In summary, this study has demonstrated that clemastine treatment following SCI enhances oligodendrocyte differentiation along with the upregulation of transcriptional factors (Myrf and Olig2), significantly motivating the ERK1/2 signaling. The mechanisms discovered from the aforementioned findings may provide a novel insight for treatment and recovery after SCI.

## Data Availability

The original contributions presented in the study are included in the article/Supplementary Material; further inquiries can be directed to the corresponding author.

## Abbreviations

Ach, acetylcholine; Cevi, cevimeline; Cle, clemastine; CNS, central nervous system; dpi, days post injury; MBP, myelin basic protein; MR, muscarinic receptor; Myrf, myelin regulatory factor; NG2, neural/glial antigen 2; OL, oligodendrocyte; Olig2, oligodendrocyte transcription factor 2; OPC, oligodendrocyte progenitor cell; P-ERK1/2, phospho-extracellular signal-regulated kinase 1/2; SCI, spinal cord injury; SD, Sprague–Dawley; T-ERK1/2, total extracellular signal-regulated kinase 1/2; WMI, white matter injury.
