# Supplementary figures and images for: Clemastine Promotes Differentiation of Oligodendrocyte Progenitor Cells Through the Activation of ERK1/2 via Muscarinic Receptors After Spinal Cord Injury
Source: Front Pharmacol. 2022 Jul 5;13:914153. doi: 10.3389/fphar.2022.914153 (PMC9294397; doi:10.3389/fphar.2022.914153)

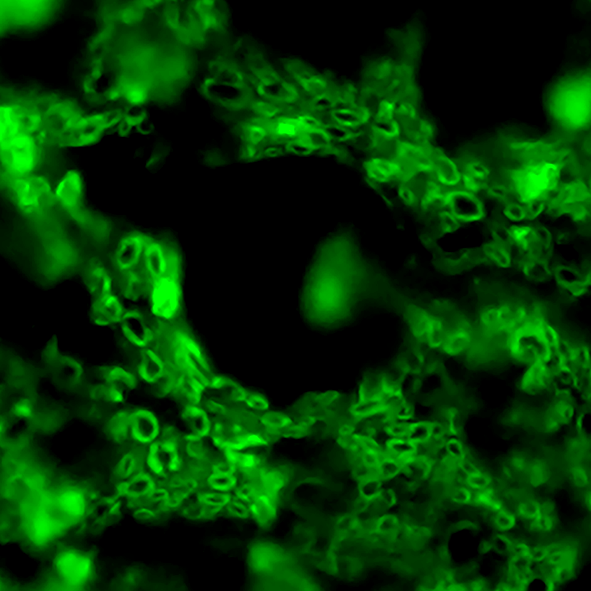

Supplement: Supplementary file 1 [file DataSheet1.ZIP › additional file(for research only/MBP14dpi-cle+cevi.tif]

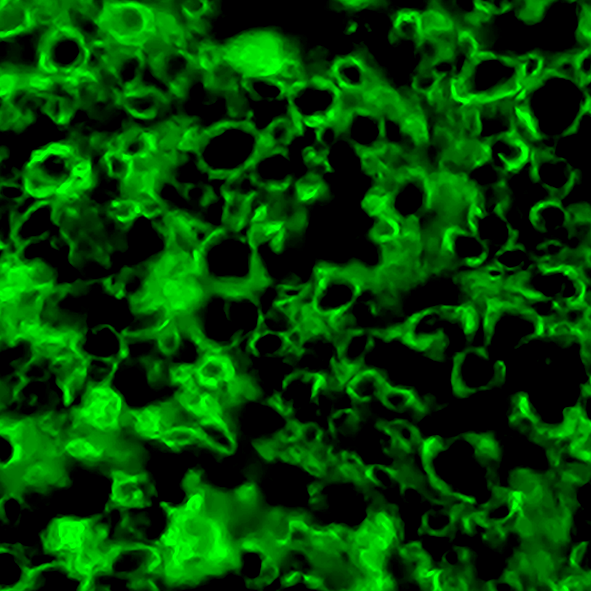

Supplement: Supplementary file 1 [file DataSheet1.ZIP › additional file(for research only/MBP14dpi-cle.tif]

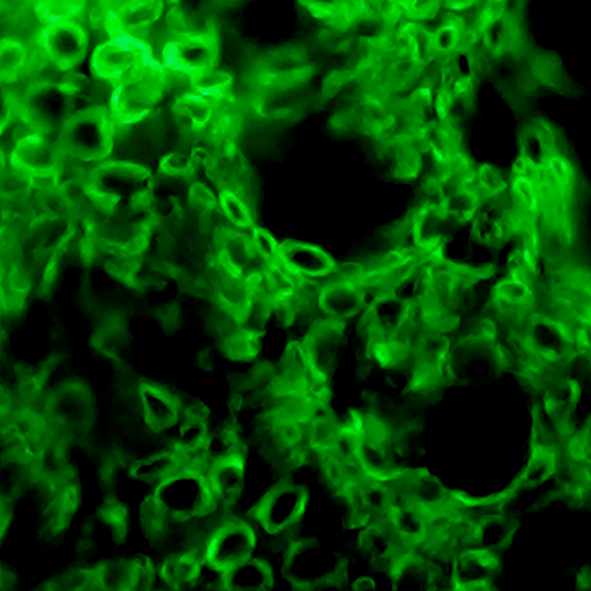

Supplement: Supplementary file 1 [file DataSheet1.ZIP › additional file(for research only/MBP14dpi-sham.tif]

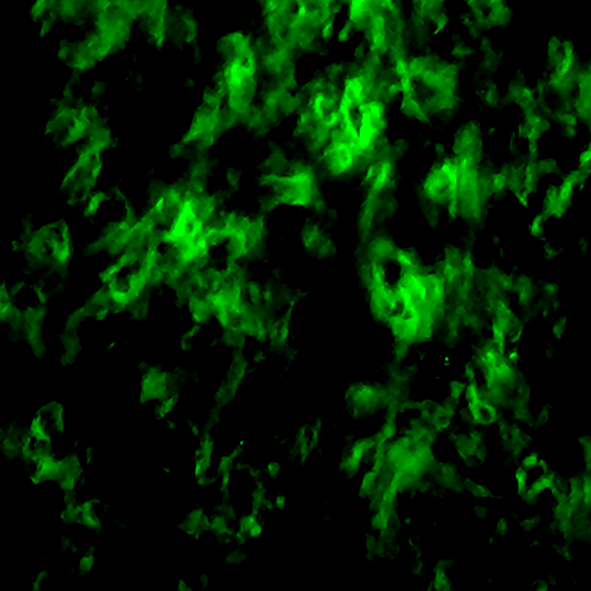

Supplement: Supplementary file 1 [file DataSheet1.ZIP › additional file(for research only/MBP14dpi-vehi.tif]

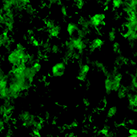

Supplement: Supplementary file 1 [file DataSheet1.ZIP › additional file(for research only/MBP7dpi-cevi.tif]

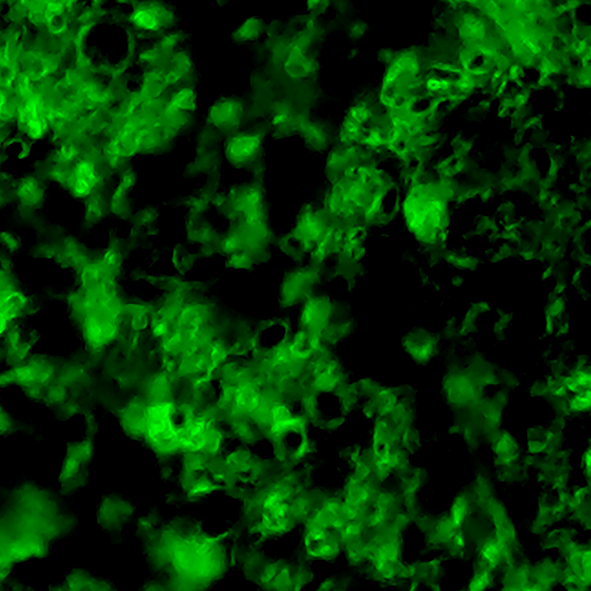

Supplement: Supplementary file 1 [file DataSheet1.ZIP › additional file(for research only/MBP7dpi-cle+cevi.tif]

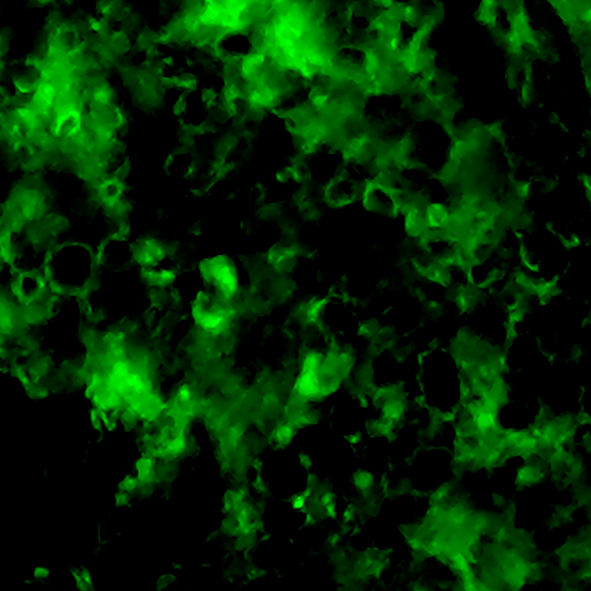

Supplement: Supplementary file 1 [file DataSheet1.ZIP › additional file(for research only/MBP7dpi-Cle.tif]

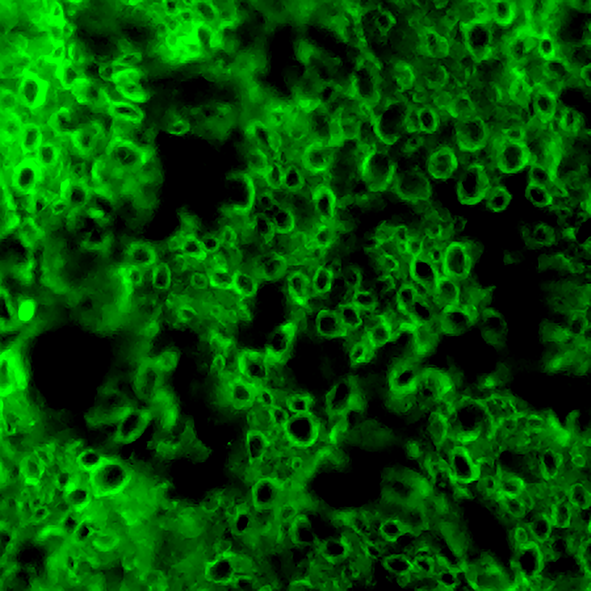

Supplement: Supplementary file 1 [file DataSheet1.ZIP › additional file(for research only/MBP7dpi-sham.tif]

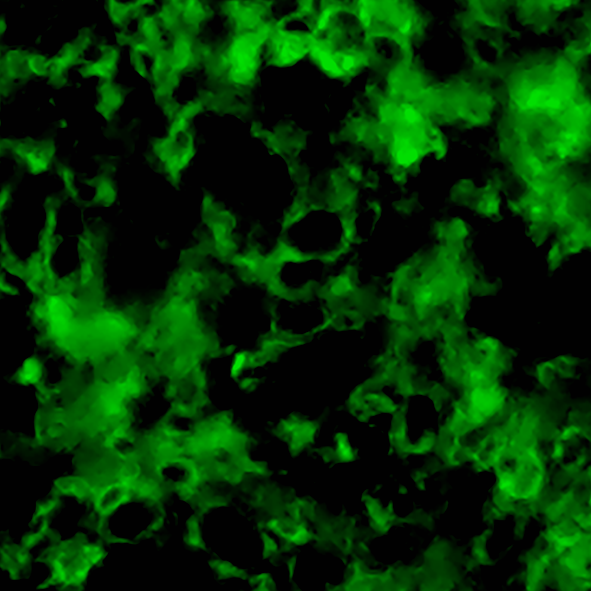

Supplement: Supplementary file 1 [file DataSheet1.ZIP › additional file(for research only/MBP7dpi-vehi.tif]

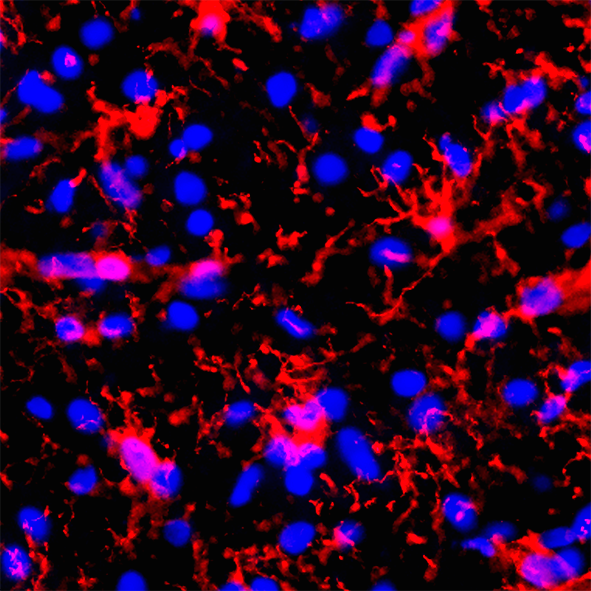

Supplement: Supplementary file 1 [file DataSheet1.ZIP › additional file(for research only/NG2-14dpi-cle+cevi.tif]

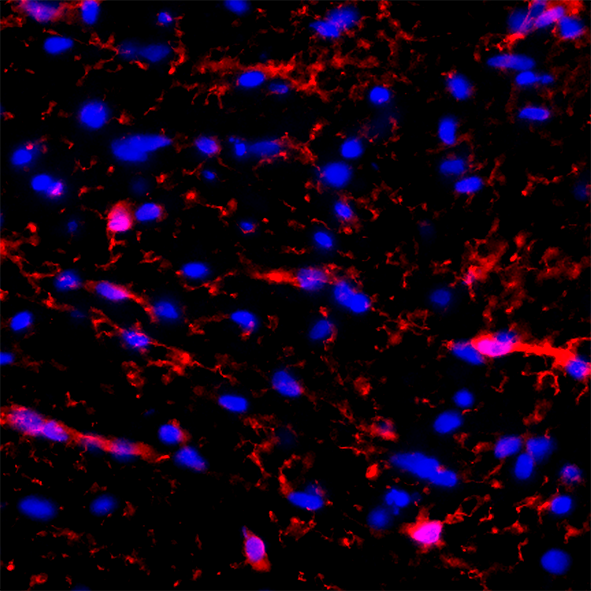

Supplement: Supplementary file 1 [file DataSheet1.ZIP › additional file(for research only/NG2-14dpi-cle.tif]

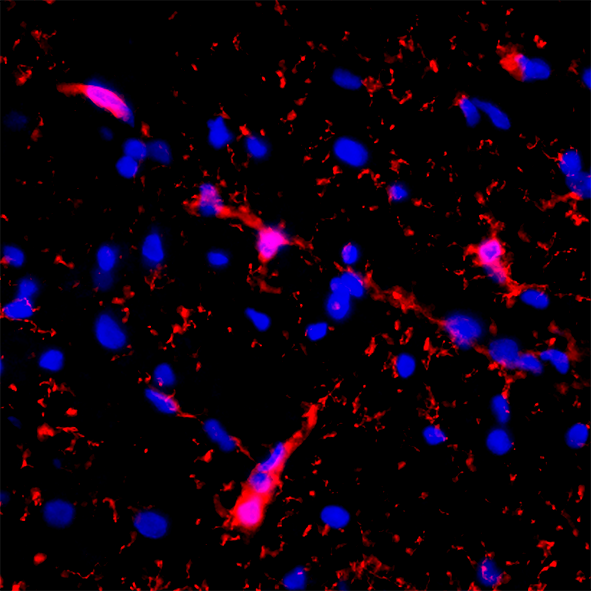

Supplement: Supplementary file 1 [file DataSheet1.ZIP › additional file(for research only/NG2-14dpi-sham.tif]

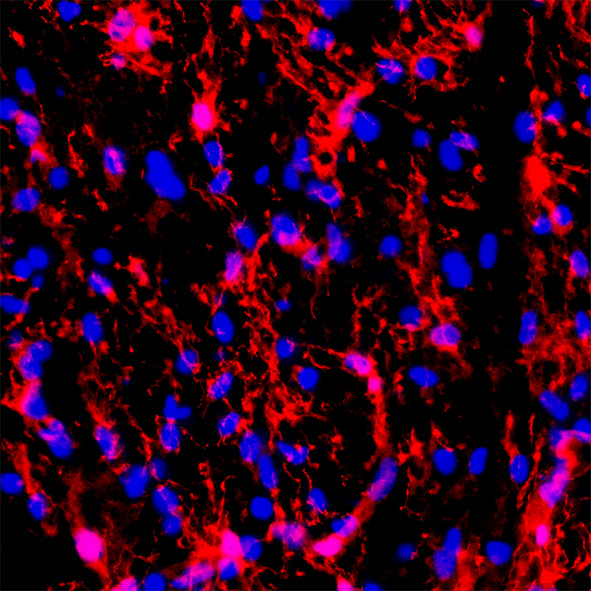

Supplement: Supplementary file 1 [file DataSheet1.ZIP › additional file(for research only/NG2-14dpi-vehi.tif]

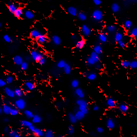

Supplement: Supplementary file 1 [file DataSheet1.ZIP › additional file(for research only/NG2-7di-cevi.tif]

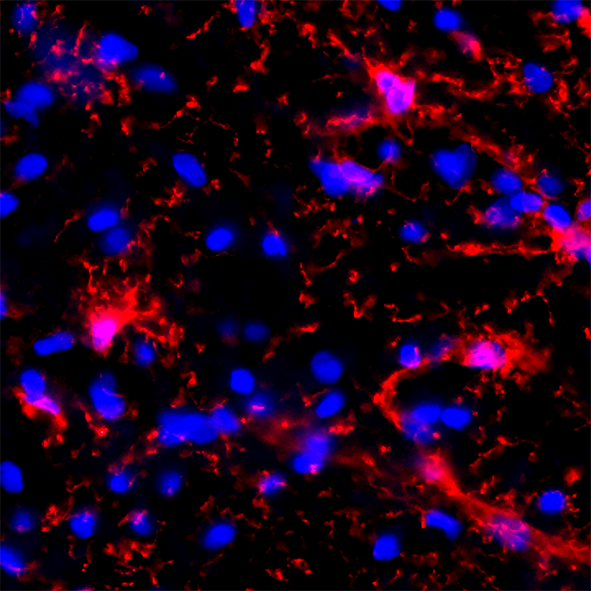

Supplement: Supplementary file 1 [file DataSheet1.ZIP › additional file(for research only/NG2-7dpi-cle+cevi.tif]

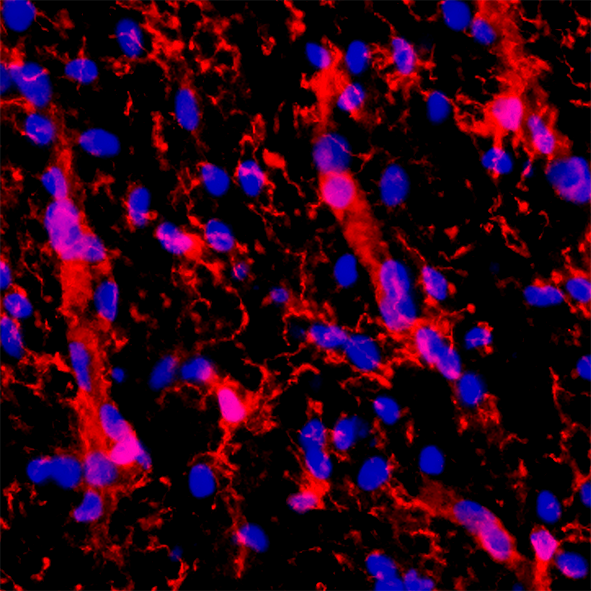

Supplement: Supplementary file 1 [file DataSheet1.ZIP › additional file(for research only/NG2-7dpi-cle.tif]

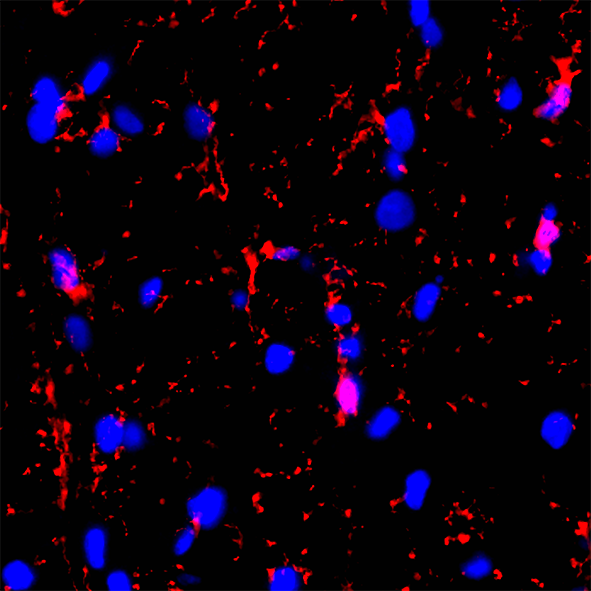

Supplement: Supplementary file 1 [file DataSheet1.ZIP › additional file(for research only/NG2-7dpi-sham.tif]

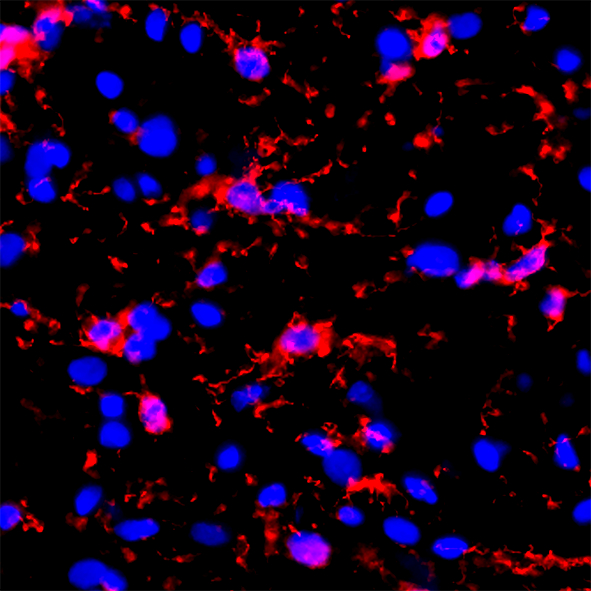

Supplement: Supplementary file 1 [file DataSheet1.ZIP › additional file(for research only/NG2-7dpi-vehi.tif]

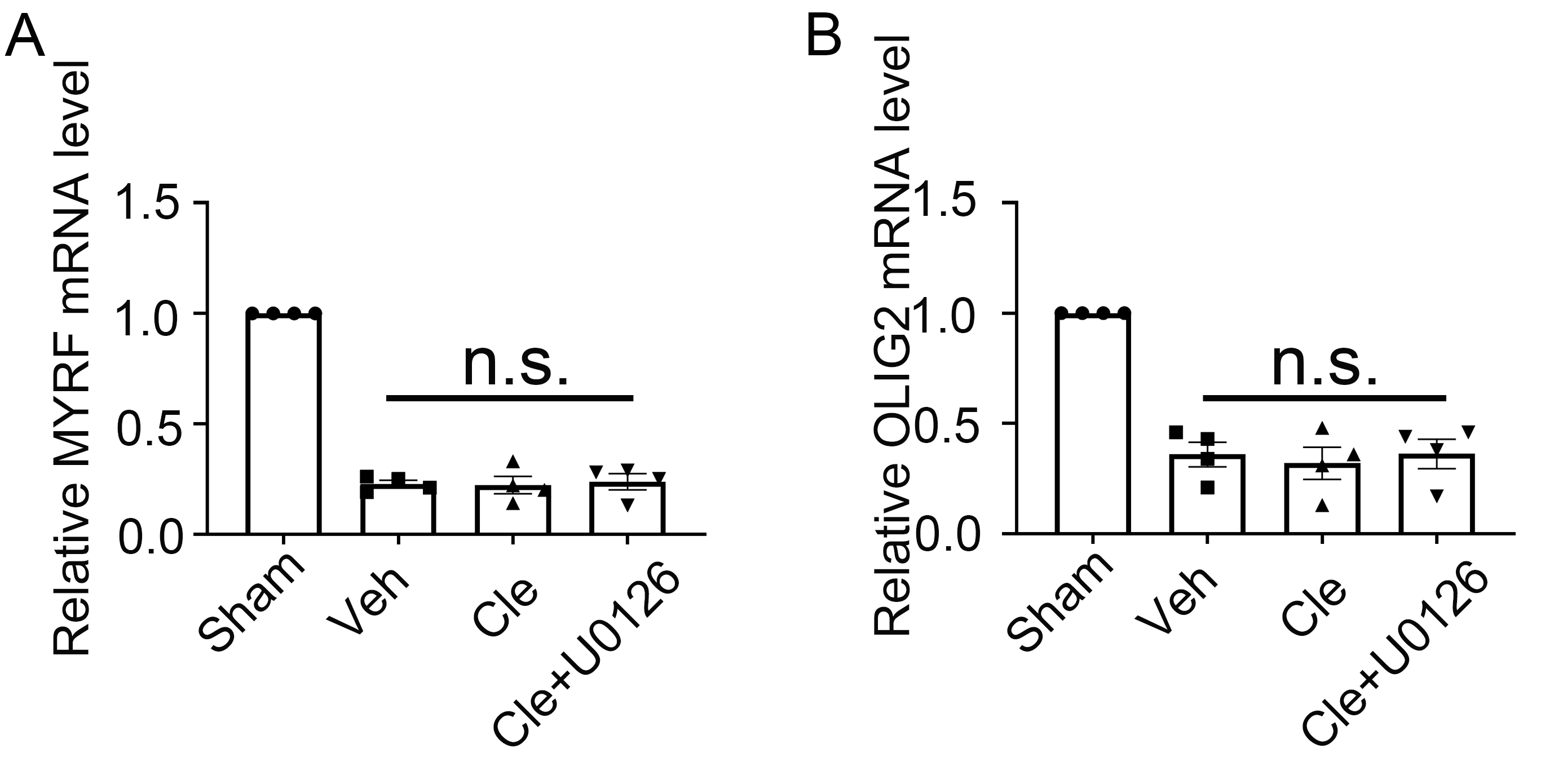

Supplement: Supplementary file 1 [file DataSheet1.ZIP › additional file(for research only/pre-test-myrf&olig2-QPCR.tif]
