# Supplementary material for: Clemastine Promotes Differentiation of Oligodendrocyte Progenitor Cells Through the Activation of ERK1/2 via Muscarinic Receptors After Spinal Cord Injury
Source: Front Pharmacol. 2022 Jul 5;13:914153. doi: 10.3389/fphar.2022.914153 (PMC9294397; doi:10.3389/fphar.2022.914153)
Supplement: Supplementary file 1 [file DataSheet1.ZIP › additional file(for research only/supplementary material-WB.zip!/supplementary material.doc]

**Figure1.**

NG2(7dpi):
MBP(7dpi):

**Figure2.**

NG2(14dpi):

MBP(14dpi):

**Figure 3:**

P-ERK/T-ERK:

P-ERK/T-ERK:
MBP(U0126):

**Figure 4:**

MYRF:

OLIG2:
